# A phase II trial of marimastat in advanced pancreatic cancer

**DOI:** 10.1054/bjoc.2001.2168

**Published:** 2001-12

**Authors:** J D Evans, A Stark, C D Johnson, F Daniel, J Carmichael, J Buckels, C W Imrie, P Brown, J P Neoptolemos

**Affiliations:** Department of Surgery, Queen Elizabeth Hospital, Birmingham, UK; Department of Surgery, Glasgow Royal Infirmary, Glasgow, UK; Plymouth Oncology Centre, Derriford Hospital, Plymouth, UK; Department of Surgery, Southampton General Hospital, Southampton, UK; CRC Department of Clinical Oncology, City Hospital, Nottingham, UK; British Biotech Pharmaceuticals Limited, Oxford, UK; University Department of Surgery, Royal Liverpool University Hospital, 5th Floor, UCD Building, Doulby Street, Liverpool, L69 36A, UK

**Keywords:** pancreatic cancer, matrix metalloproteinases, marimastat, toxicity

## Abstract

Pancreatic cancer has a poor response to conventional chemotherapy and radiotherapy. Inhibition of matrix metalloproteinase activity involved in tumour invasion and metastases is a novel biological approach for cancer treatment. This multicentre phase II clinical trial assessed marimastat, an oral matrix metalloproteinase inhibitor, in patients with advanced pancreatic cancer. A total of 113 patients received marimastat for 28 days at 100 mg b.d. (*n* = 9), 25 mg o.d. (*n* = 90) or 10 mg b.d. (*n* = 14). Patients with a response to treatment could continue marimastat beyond 28 days. Of 113 patients, 90 (80%) completed the 28-day study and 83 (73%) continued treatment. The principal side effect was arthralgia in 14 (12%) patients at 28 days and 33 (29%) patients over the whole study. There were 31 patients (27%) who required dose modification. Of 76 patients with evaluable CA19-9 levels, 23 (30%) showed no increase or fall in CA19-9. Of 83 patients with radiologically assessable disease, 41 (49%) had stable disease. The median survival was 245 days for those with a stable or falling CA19-9 level 128 days in those with rising CA19-9. The overall survival was 3.8 months. 5.9 months for stage II, 4.7 months for stage III and 3 months for stage IV disease. Of 90 patients, 46 (51%) had stabilization or reduction in pain, mobility and analgesia scores. Further development and clinical evaluation of matrix metalloproteinase inhibitors for the treatment of pancreatic cancer is warranted. © 2001 Cancer Research Campaign http://www.bjcancer.com


*British Journal of Cancer* (2001) **85**(12), 1865–1870
© 2001 Cancer Research Campaign
doi: 10.1054/ bjoc.2001.2168, available online at http://www.idealibrary.com on IDEAL

http://www.bjcancer.com

### A phase II trial of marimastat in advanced pancreatic cancer

**JD Evans[1], A Stark[2], CD Johnson[3], F Daniel[4], J Carmichael[5], J Buckels[1], CW Imrie[2], P Brown[6] and JP Neoptolemos[1,7]**

[1]Department of Surgery, Queen Elizabeth Hospital, Birmingham, UK; [2]Department of Surgery, Glasgow Royal Infirmary, Glasgow, UK; [3]Plymouth Oncology Centre, Derriford Hospital, Plymouth, UK; [4]Department of Surgery, Southampton General Hospital, Southampton, UK; [5]CRC Department of Clinical Oncology, City Hospital, Nottingham, UK; [6]British Biotech Pharmaceuticals Limited, Oxford, UK; [7]University Department of Surgery, Royal Liverpool University Hospital, 5th Floor, UCD Building, Doulby Street, Liverpool L69 36A, UK

**Summary** Pancreatic cancer has a poor response to conventional chemotherapy and radiotherapy. Inhibition of matrix metalloproteinase activity involved in tumour invasion and metastases is a novel biological approach for cancer treatment. This multicentre phase II clinical trial assessed marimastat, an oral matrix metalloproteinase inhibitor, in patients with advanced pancreatic cancer. A total of 113 patients received marimastat for 28 days at 100 mg b.d. ($n$ = 9), 25 mg o.d. ($n$ = 90) or 10 mg b.d. ($n$ = 14). Patients with a response to treatment could continue marimastat beyond 28 days. Of 113 patients, 90 (80%) completed the 28-day study and 83 (73%) continued treatment. The principal side effect was arthralgia in 14 (12%) patients at 28 days and 33 (29%) patients over the whole study. There were 31 patients (27%) who required dose modification. Of 76 patients with evaluable CA19-9 levels, 23 (30%) showed no increase or fall in CA19-9. Of 83 patients with radiologically assessable disease, 41 (49%) had stable disease. The median survival was 245 days for those with a stable or falling CA19-9 level 128 days in those with rising CA19-9. The overall survival was 3.8 months. 5.9 months for stage II, 4.7 months for stage III and 3 months for stage IV disease. Of 90 patients, 46 (51%) had stabilization or reduction in pain, mobility and analgesia scores. Further development and clinical evaluation of matrix metalloproteinase inhibitors for the treatment of pancreatic cancer is warranted. © 2001 Cancer Research Campaign http://www.bjcancer.com

**Keywords**: pancreatic cancer; matrix metalloproteinases; marimastat; toxicity

Pancreatic cancer is an aggressive disease with an overall 5-year survival rate of 0.4% (Bramhall et al, 1995). Most patients present with advanced disease with only 2.6–4% undergoing resection (Bramhall et al, 1995; NYCRIS, 2000). In advanced disease, the median survival is only 4–6 months and over 90% of patients are dead within 1 year. Chemotherapy and radiotherapy are associated with substantial toxicity and confer only a marginal improvement in survival (Bakkevold, 1998).

The matrix metalloproteinases (MMPs) are a family of at least 18 proteolytic enzymes that can be subdivided into four main groups according to substrate specificity, namely: the collagenases, stromelysins, gelatinases (type IV collagenases) or membrane-bound MMPs (MT-MMPs). Collectively, they are able to degrade all components of the extracellular matrix (Jones et al, 1999). MMP activity is regulated by four specific tissue inhibitors of the metalloproteinases (TIMPs) (Greene et al, 1996; Sellers et al, 1997; Stetler-Stevenson, 1989; Uria et al, 1994). MMPs have an important physiological role in embryonic development (Brenner et al, 1989), bone remodelling (Delaisse and Vaes, 1992) and wound healing (Wolf et al, 1992), and a major role in pathological processes including rheumatoid arthritis (Harris, 1990) and cancer invasion and metastasis (Liotta and Stetler-Stevenson, 1991). MMP overexpression has been reported in carcinomas of the ovary (Campo et al, 1992), prostate (Pajouh et al, 1991) and lung (Brown et al, 1993) and MMP activity correlated with malignant potential in gastric (Nomura et al, 1995) and colorectal (Mori et al, 1995; Porte et al, 1995) cancer.

Several studies have indicated that MMP and TIMP expression are also deranged in pancreatic cancer. Bramhall et al reported overexpression of MMPs 2 and 3 with TIMP 1 in pancreatic and ampullary cancers (Bramhall et al, 1996). Subsequent studies demonstrated that MMPs 2, 7 and 9 and TIMPs 1 and 2 are those most abundantly expressed in pancreatic cancer tissue with MMP2 and MT-MMP1 co-expressed in 60% of pancreatic cancers (Bramhall et al, 1997; Gress et al, 1995). TIMP1 expression has been shown to have an inverse relationship with the presence of lymph node metastases while increased MMP expression is associated with tumour de-differentiation (Bramhall et al, 1994, 1996). More recently, overexpression of MMPs 2, 7, 8, 9, 12 with reduced expression of TIMPs 1 and 2 (Jones et al, 2000) has been reported.

These findings have led to the development of a number of synthetic matrix metalloproteinase inhibitors (MMP1s). Marimastat (British Biotech, Oxford, UK) was the first orally bioavailable broad-spectrum MMPI to enter clinical trials. Structurally, marimastat contains a hydroxyamate group that chelates the zinc atom at the active site of the MMPs, resulting in reversible inhibition of enzyme activity. Marimastat is active against all major classes of MMPs and displays an $IC_{50}$ of 5 nM, 6 nM, 3 nM and 200 nM against collagenase, gelatinase 72 kDa and 92 kDa and stromelysin, respectively, at nanomolar concentrations (Beckett et al, 1996). Phase I studies demonstrated good tolerability with an elimination half-life of 8–12 h. A linear relationship was shown between dose and plasma concentration with high plasma levels achieved following a single oral

*Received 10 April 2001*
*Accepted 03 October 2001*

*Correspondence to:* JP Neoptolemos daily dose (Drummond et al, 1995). The aim of this study was to evaluate the effect and define the tolerability of marimastat in patients with advanced pancreatic cancer.

### MATERIALS AND METHODS

### Study design

This was a multicentre open phase II pilot clinical trial to assess the effect of 28 days' administration of oral marimastat (British Biotech Pharmaceuticals Ltd, Oxford, UK) in patients with advanced pancreatic cancer with a facility to continue treatment in those patients with a clinical, serological or radiological 'response' to treatment. Marimastat was administered at a dose of 100 mg b.d., 25 mg o.d. or 10 mg b.d. with the option to reduce the dose in the event of toxicity. Patients were recruited from five centres throughout the UK between August 1995 and December 1997. Ethical committee approval was obtained from the research ethics committee at each investigating centre prior to study commencement.

### Study objectives

The aims of this study were:

1. To evaluate the effect of 28 days' treatment with oral marimastat on tumour size measured by computerize tomography (CT), disease progression was assessed by the level of cancer antigen CA19-9 and pain, mobility and analgesic use scores
2. To define the tolerability of marimastat
3. To assess the tumour response, safety and tolerability in patients treated beyond 28 days.

### Patient selection

Patients with radiologically and/or histologically/cytologically proven advanced pancreatic cancer were considered for study entry. Inclusion criteria were: (1) patients over 18 years of age; (2) Eastern Cooperative Oncology Group (ECOG) performance status of 0–2; (3) estimated survival of at least 3 months and the ability to take food by mouth. Exclusion criteria included: (1) previous systemic antineoplastic therapy or radiotherapy within 2 months of study entry; (2) serum bilirubin level > 100 μmol/l; (3) serum albumin level < 25 g/l or positive serology for HIV or HBsAg. Pre-menopausal females had a negative pregnancy test prior to study entry. Written informed consent was obtained from all patients and all patients were commenced on pancreatic enzyme supplements at study entry.

### Clinical assessment of patients

Patients entered one of three initial screening periods according to their presentation:

1. Patients who had newly diagnosed pancreatic cancer and did not require stenting or surgery (group 1) were screened 1 week prior to study entry
2. Patients with newly diagnosed pancreatic cancer who required stenting or surgery (group 2) were screened at least 3 weeks prior to entry
3. Those with serologically detected recurrent cancer (group 3) were screened at least 6 weeks prior to study entry, requiring that the serum CA19-9 level was at least twice the upper limit of normal on three consecutive occasions or a 20% increase over a 6-week period. The stage of disease was assessed prior to study entry according to the American Joint Committee for Cancer (AJCC) staging system for pancreatic cancer (Cancer of the Pancreas Task Force, 1981).

Following study entry, patients were assessed at days 0, 7, 14 and 28, and monthly thereafter in those patients who continued treatment. Patients who did not continue treatment had a final assessment on day 56. A full history and clinical examination was performed 1 week prior to study entry and repeated at day 28 and monthly on subsequent visits. Concomitant medication, invasive tests, vital signs (pulse, blood pressure, oral temperature, respiratory rate) and a clinical assessment of pain, mobility ECOG score (Smith et al, 1980) and analgesic use (graded as: 0 = none; 1 = simple analgesic or non-steroidal anti-inflammatory drugs (NSAIDS); 2 = simple analgesic plus NSAIDS; 3 = moderate analgesia such as co-proxamol; 4 = opiates < 40 mg morphine (or equivalent) daily; 5 = opiates > 40 mg but < 100 mg morphine daily; 6 = opiates > 100 mg morphine daily) was recorded at each visit.

Routine haematology, biochemistry including C-reactive protein (CRP) and urinalysis was performed 1 week prior to study entry, on days 0, 7 and 28 and on each subsequent visit. Serial electrocardiograms were performed prior to study entry, at day 28 and monthly thereafter. Serum CA19-9 was measured at 6, 4 and 2 weeks before study entry in patients with serologically detected recurrent disease (group 3), 3 and 2 weeks prior to entry in group 2 and 1 week prior to entry in group one. All patients then had CA19-9 measurements on days 0, 7, 14 and 28, and then monthly. Contrast-enhanced spiral CT scanning (CE-CT) was performed within 2 weeks of study entry, at day 28 then 2, 4, 8 and 12 months after study entry according to a standardized protocol and reported by a single independent radiologist in each study centre. Adverse and serious adverse events were reported immediately to British Biotech Pharmaceuticals Ltd.

### Definition of clinical response

A clinical response to treatment was defined as one or more of: (1) a serum CA19-9 level at day 28 less than or equal to the level at day 0 (serological response); (2) complete or partial response or the presence of stable disease on CT scan (radiological response) using WHO criteria or a pain, mobility and analgesia score that was no greater at day 28 than at day 0 (clinical response).

### Pharmacokinetic assay

Samples of venous blood were collected immediately before and 2 hours after the first administration of marimastat on day zero, prior to the morning dose on days 14 and 28, and monthly thereafter. Additional samples were taken when any serious adverse event was suspected and considered to be drug related. Samples were immediately put on ice and within 30 min of collection, plasma extracted and stored at –70˚C until analysis was carried out in a central laboratory.

### Statistical analysis

Data was analysed using Kaplan–Meier survival curves and the log rank (Mantel–Cox) statistical test.

 *© 2001 Cancer Research Campaign*

### RESULTS

### Patient characteristics

113 patients with advanced pancreatic cancer were entered into the study. The median age was 63 (range 32–84) years; 72 (64%) patients were men. Of the patients included in the study, 28 (25%) patients had stage II disease, 21 (19%) had stage III disease and 64 (57%) had stage IV disease. The median time between diagnosis and study entry was 51 days. There were 91 patients (81%) who had previously undergone surgery ranging from diagnostic laparoscopy to a pancreatico-duodenectomy. Nine patients (8%) had previously received 5-FU-based chemotherapy. A total of 64 patients (57%) had a histological diagnosis of pancreatic ductal adenocarcinoma, 2 (2%) had adenosquamous carcinomas and 1 (1%) had small cell carcinoma of the pancreas. No histological confirmation was available in 46 patients (41%). Two patients (2%) had co-existent bladder and breast cancer, respectively.

**Table 1** Change in pain, analgesic use and WHO performance score from days 0–28 (*n* = 88)

| Dose | Increase | No change | Decrease |
|---|---|---|---|
| | Change in pain score | | |
| 25 mg o.d. | 19 | 40 | 12 |
| 10 mg o.d. | 4 | 1 | 4 |
| 100 mg b.d. | 2 | 5 | 1 |
| Total | 25 (28%) | 46 (52%) | 17 (19%) |
| | Change in analgesic use score | | |
| 25 mg o.d. | 17 | 47 | 7 |
| 10 mg o.d. | 2 | 7 | 0 |
| 100 mg b.d. | 0 | 6 | 2 |
| Total | 19 (22%) | 60 (68%) | 9 (10%) |
| | Change in WHO performance score | | |
| 25 mg o.d. | 11 | 55 | 5 |
| 10 mg o.d. | 1 | 6 | 2 |
| 100 mg b.d. | 0 | 8 | 0 |
| Total | 12 (14%) | 69 (78%) | 7 (8%) |

### Treatment dosage and duration

The first nine patients were commenced on marimastat 100 mg b.d. Early pharmacokinetic data from these patients indicated that a dose reduction was necessary and a protocol amendment resulted in a reduction of the dose to 25 mg o.d. A further protocol amendment introduced a third dose of 10 mg b.d. in light of other ongoing studies on marimastat, which suggested that this was the optimum dosage regime. A total of 90 patients (80%) completed the main 28-day study period and 83 (73%) continued treatment. Median duration of treatment was 78, 60 and 80 days for those starting treatment at 25 mg o.d. (*n* = 90), 10 mg b.d. (*n* = 14) and 100 mg b.d. (*n* = 9), respectively (range 1–486 days) including interruptions for tolerability reasons.

### Response to treatment

Of 76 patients with evaluable CA19-9 levels, 23 (30%) showed stabilization or a fall in levels during the 28-day study period; 4 patients had received 100 mg b.d. and 19 had received 25 mg o.d. Of the 83 patients with radiologically assessable disease, 41 (49%) had stable disease over the initial 28-day study period, while 42 (51%) showed evidence of disease progression. Of 41 patients with radiologically stable disease, 13 (32%) had stable or reduced CA19-9 levels, 24 (58%) had a rise in CA19-9 and 4 (10%) had non-evaluable CA19-9 levels. In those with radiologically progressive disease, 9 (21%) of 42 patients had stable or reduced CA19-9 levels, 26 (62%) had a rise in CA19-9 and seven (17%) had non-evaluable CA19-9 levels. 46 (51%) of 90 patients had a decrease or stabilization of combined pain, mobility and analgesia scores from day 0 to day 28 (Table 1). Seven patients (8%) met all three of requirements to classify them as having a response to treatment.

The overall median survival for the 113 patients who entered the study was 113 days. Survival was 5.9, 4.7 and 3 months for stages II, III and IV disease respectively ($P < 0.04$; Figure 1). The median survival for those with a fall or stabilization of CA19-9 levels was 245 days vs 128 days for those with a rising CA19-9 level ($P = 0.004$; Figure 2). Patients were also subdivided into two groups according to the rate of rise in CA19-9 levels over the 28-day study period. Group one was made up of those (*n* = 52) in whom the rate of CA19-9 was greater than the median and group two included those (*n* = 52) in whom CA19-9 levels rose by less than or equal to the median. Those patients who had a lower rate of rise in CA19-9 had a prolonged median survival time of 183 days compared with 90 days in the remainder of patients ($P < 0.003$). There was no statistically significant difference in survival between those patients with radiologically stable disease compared with those with progressive disease at 28 days ($P = 0.20$).

### Pharmacokinetics

Trough plasma marimastat levels were markedly higher in patients receiving 100 mg b.d. (mean 321.7 μg/l, range 133–601 μg/l) than those receiving 25 mg o.d. (mean 45.2 μg/l, range 6–177 μg/l) or 10 mg b.d. (mean 57.5 μg/l, range 34–109 μg/l). Steady state plasma trough levels were therefore higher in patients with advanced pancreatic cancer when compared with healthy volunteers. Mean plasma trough levels of marimastat following a once daily dosing regimen, however, were well above the concentration required to inhibit the major MMPs implicated in tumour invasion.

### Adverse events

The adverse events reported during the 28-day study period are shown in Table 2. Over the total study period, musculoskeletal side effects were increasingly common with arthralgia experienced by 33 (29%) patients, back pain in 10 (9%), joint stiffness in 10 (9%) and myalgia in 11 (10%). Marimastat treatment was interrupted in 31 patients (27%) who continued treatment beyond 28 days because of musculoskeletal side effects (Table 3). Serious adverse events were reported in 104 patients (92%). Serious adverse events (*n* = 11) with possible relation to marimastat were reported in 9 patients (8%) including disseminated carcinoma (*n* = 5), anaemia (*n* = 1), diarrhoea (*n* = 1), nausea (*n* = 1), jaundice (*n* = 1), gastrointestinal bleeding (*n* = 1) and myocardial infarction (*n* = 1). The reported outcomes in 4 of the 5 cases of disseminated disease and the patient with myocardial infarction were death. All serious adverse events were considered to be disease related.

*© 2001 Cancer Research Campaign* 

**Table 2** Common adverse events considered to be at least possible related to treatment

| Dose | 25 mg o.d. | 10 mg b.d. | 100 mg b.d. | Total |
|---|---|---|---|---|
| | Days 0–28 | | | |
| No. treated | 90 | 14 | 9 | 113 |
| With related adverse events | 32 (35.6%) | 4 (28.6%) | 7 (77.8%) | 43 (38.1%) |
| Musculoskeletal | | | | |
| Arthralgia | 9 | 3 | 2 | 14 (12.4%) |
| Myalgia | 4 | 2 | 0 | 6 ( 5.3%) |
| | Total study period | | | |
| No. treated | 90 | 14 | 9 | 113 |
| With related adverse events | | | | |
| Musculoskeletal | | | | |
| Arthralgia | 22 | 7 | 4 | 33 (29.2%) |
| Back pain | 7 | 2 | 1 | 10 ( 8.8%) |
| Joint stiffness | 8 | 1 | 1 | 10 ( 8.8%) |
| Myalgia | 6 | 3 | 2 | 11 ( 9.7%) |
| Pain in limb | 6 | 1 | 0 | 7 ( 6.2%) |
| Skeletal pain | 4 | 0 | 2 | 6 ( 5.3%) |
| Gastrointestinal | | | | |
| Dyspepsia | 5 | 1 | 0 | 6 ( 5.3%) |
| Nausea | 7 | 0 | 2 | 9 ( 8.0%) |
| Vomiting | 6 | 0 | 0 | 6 ( 5.3%) |

**Table 3** Patients withdrawn or dose modified due to musculoskeletal side effects

| Dose | No. recruited | No. continuing | Patient withdrawn or dose modified (number) | | | |
|---|---|---|---|---|---|---|
| | | | Days 0–28 | Days 56 | Days 0–200 | Days 0–300 |
| 25 mg o.d. | 90 | 67 | 4 | 7 | 19 | 21 |
| 10 mg o.d. | 14 | 8 | 1 | 4 | 4 | 5 |
| 100 mg o.d. | 9 | 8 | 2 | 4 | 5 | 5 |

### DISCUSSION

The prognosis for patients with pancreatic cancer remains very poor. There have been many trials on the value of chemotherapy and radiotherapy in advanced pancreatic cancer but the results have generally been disappointing. Three out of four randomized, controlled trials have shown a benefit for chemotherapy compared to no active treatment or best supportive care. Frey et al found no survival benefit using a combination of 5-FU and carmustine (Frey et al, 1980). Mallison et al, however, demonstrated a median survival of 11 months using a combination of 5-FU, cyclophosphamide, methotrexate, vincristine and mitomycin C compared with 2.2 months for the untreated control group (Mallinson et al, 1980). Studies using 5-FU, doxorubicin and mitomycin C (FAM) and a combination of 5-FU, folinic acid and etoposide have similarly shown improved survival together with a better quality of life (Smith et al, 1980; Palmer et al, 1994; Glimelius et al, 1996). More recently, gemcitabine was shown in a randomized trial to increase median survival time to 5.7 months compared to 4.4 months in patients given bolus 5-FU (Burris et al, 1997). Moreover, there was improved 1 year survival rate with gemcitabine (22% vs 2%) and a significantly better clinical benefit response.

The resistance of pancreatic cancer to conventional chemotherapy and radiotherapy has driven the development of novel therapeutic agents such as the MMPIs. This study has demonstrated that marimastat is well absorbed following oral administration with therapeutic plasma levels achieved at a dose of 25 mg o.d. The principal adverse effect attributable to marimastat was musculoskeletal side effects that had a variable presentation from joint pain and stiffness, tendinitis and myalgia. Musculoskeletal side effects were observed in 14% of patients by 28 days' treatment but the incidence was clearly dose and time dependent, with one-third of patients treated beyond 28 days suffering significant musculoskeletal symptoms necessitating interruption of treatment and/or dose reduction. The precise mechanism by which marimastat causes musculoskeletal side effects is not entirely understood but may be due to disruption of the normal balance between MMPs and TIMPs within and around joints. The future development of more specific MMPIs that affect only the MMPs important in pancreatic cancer progression, may enable musculoskeletal side effects to be minimized.

Of 76 patients with evaluable cancer antigen levels, 23 (30%) showed a fall or no change in CA19-9 levels during the 28-day study period. This 'serological response' to treatment was associated with a significant improvement in survival (245 days vs 128 days) and patients with a lower rate of rise in CA19-9 also had a prolonged median survival time (183 days vs 90 days). Whilst this indicates that CA19-9 may be a useful measure of treatment response, this tumour marker is not ideal. CA19-9 was only expressed in 75% of patients with pancreatic cancer, and 9 (21%) of 42 patients with radiologically progressive disease in this study had stable or reduced CA19-9 levels. These findings demonstrate the limitations of CA19-9 as a surrogate market of treatment response. Furthermore improved radiological appearances following treatment did not translate into improved survival.

 *© 2001 Cancer Research Campaign*

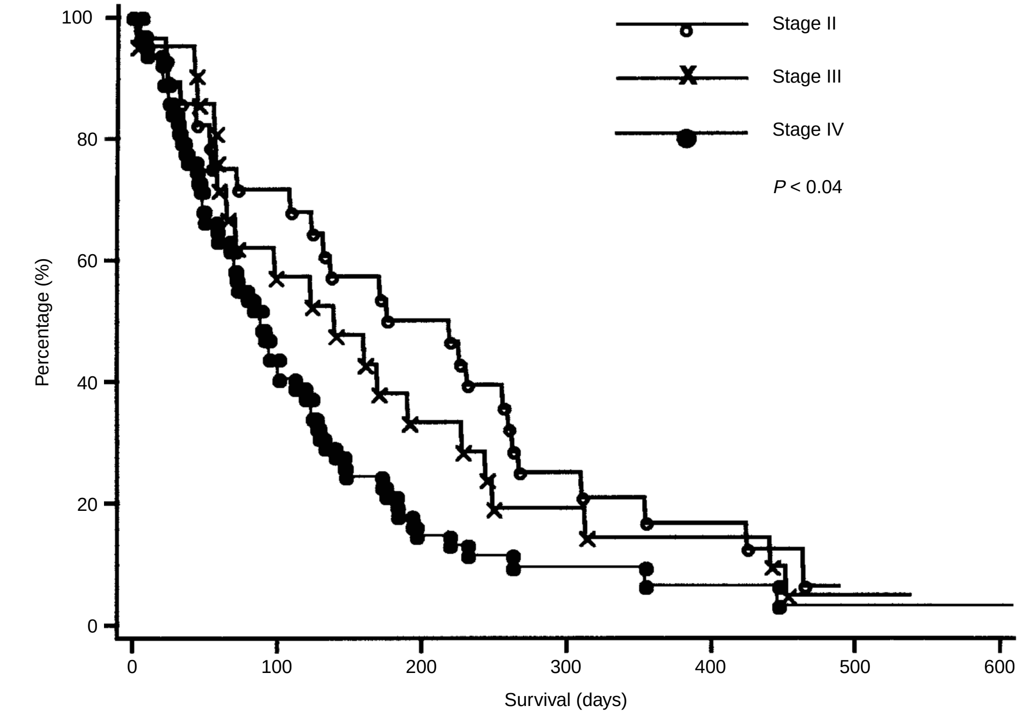

| | 0 | 100 | 200 | 300 | 400 | 500 | 600 | |
|---|---|---|---|---|---|---|---|---|
| | 28 | 20 | 14 | 6 | 4 | 0 | 0 | stage 2 |
| (at risk) | 21 | 12 | 7 | 4 | 3 | 1 | 1 | stage 3 |
| | 64 | 27 | 9 | 5 | 2 | 1 | 1 | stage 4 |

**Figure 1** Kaplan–Meier plot demonstrating survival according to stage of disease at study entry.

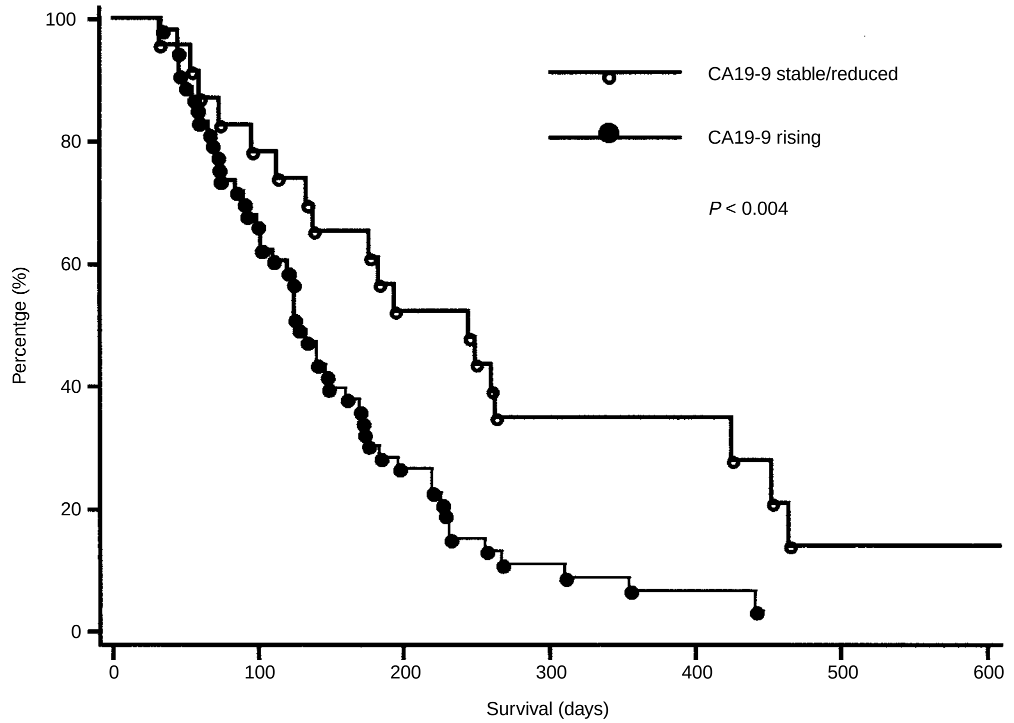

| | 0 | 100 | 200 | 300 | 400 | 500 | 600 | |
|---|---|---|---|---|---|---|---|---|
| (at risk) | 23 | 18 | 12 | 7 | 5 | 1 | 1 | CA19-9 stable |
| | 55 | 35 | 14 | 5 | 3 | 0 | 0 | CA19-9 rising |

**Figure 2** Kaplan–Meier plot demonstrating survival according to CA19-9 response to treatment.

*© 2001 Cancer Research Campaign*

Median survival time in this study was only 113 days (3.8 months), which may reflect patient selection and bias towards more advanced disease. Marimastat was associated with stabilization or reduction in pain, mobility and analgesic use scores, similar criteria to those used to assess clinical benefit response in the gemcitabine study (Burris et al, 1997) in half of the patients who entered the study. More recently a pivotal randomized, controlled study comparing marimastat against gemcitabine in 414 patients with non-resectable pancreatic cancer demonstrated no difference in survival rate in patients receiving 25 mg b.d. marimastat compared with those treated with once weekly i.v. gemcitabine at 1000 mg/m$^2$. No difference in quality of life scores or treatment-related adverse events was demonstrated and this study is continuing (Bramhall et al, 2001).

In clinical terms, marimastat appears to be a tumouristatic rather than a cytotoxic agent. Maximal benefit would be expected in patients with minimal or microscopic residual disease and randomized, controlled trials are now in progress to evaluate the effect of marimastat in the adjuvant setting following resection of pancreatic cancer. Further development and clinical evaluation of MMPIs in pancreatic cancer is warranted.

### REFERENCES

Bakkevold KE (1998) Chemotherapy for unresectable pancreatic cancer. In: Beger H, Warshaw AL, Buchler MW, Carr-Locke DL, Neoptolemos JP, Russell C, Sarr MG (eds), *The Pancreas,* pp 1120–1138. Blackwell Science Oxford

Beckett RP, Davison AH, Drummond AH, Huxley P and Whittaker M (1996) Recent advances in matrix metalloproteinase inhibitor research. *Drug Dev Today* **1**: 16–26

Bramhall SR, Lemoine N, Stamp G, Donovan IA, Dunn J and Neoptolemos JP (1994) 72kDa collagenase, Stromelysin 1 and tissue inhibitor of metalloproteinase 1 expression in pancreatic cancer. *Digestion* **55**: 286

Bramhall SR, Allum WH, Cummings C, Jones AG, Attwood A and Neoptolemos JP (1995) Incidence, treatment and outcome in 13,600 cases of pancreatic cancer: An epidemiological study in the West Midlands. *Br J Surg* **82**: 111–115

Bramhall SR, Stamp G, Dunn J, Lemoine N and Neoptolemos JP (1996) Expression of collagenase (MMP2), stromelysin (MMP3) and tissue inhibitor of metalloproteinases (TIMP1) in pancreatic and ampullary disease. *Br J Cancer* **73**: 972–978

Bramhall SR, Lemoine N, Stamp G and Neoptolemos JP (1997) Imbalance of expression of matrix metalloproteinases (MMPs) and tissue inhibitors of the matrix metalloproteinases (TIMPs) in human pancreatic cancer. *J Pathol* **182**: 347–355

Bramhall SR, Rosemurgy A, Brown PD, Bowry C and Buckels JA (2001) Marimastat as first line therapy for patients with unresectable pancreatic cancer: a randomized trial. *J Clin Oncol* **19**: 3447–3455

Brenner DA, Ohara M, Angel P, Chojkier M and Karin M (1989) Prolonged activation of jun and collagenase genes by tumour necrosis factor alpha. *Nature* **337**: 661–663

Brown PD, Bloxidge RE, Stuart NSE, Gatter KC and Carmichael J (1993) Association between expression of activated 72-kilodalton gelatinase and tumour spread in non-small cell lung carcinoma. *J Natl Cancer Inst* **85**: 574–578

Burris HA, Moore MJ, Andersen J, Green M, Rothenberg ML, Modiano MR, Cripps MC, Portenoy RK, Torniolo AM, Tarassoff P, Nelson R, Dorr FA, Stephens CD and Von Hoff DD (1997) Improvements in survival and clinical benefit with gemcitabine as first-line therapy for patients with advanced pancreatic cancer: a randomized trial. *J Clin Oncol* **15**: 2403–2413

Campo E, Merino MJ, Tavassoli FA, Charonis AS, Stetler-Stevenson WG and Liotta LA (1992) Evaluation of basement membrane components and the 72kDa type IV collagenase in serous tumours of the ovary. *Am J Surg Pathol* **16**: 500–507

Cancer of the Pancreas Task Force (1981) Staging cancer of the pancreas. *Cancer* **47**: 1631–1642

Delaisse JM and Vaes G (1992) Mechanism of mineral solublisation and matrix degradation in osteoclastic bone resorption. In: Rifkin BR and Gay CV (eds), *Biology and Physiology of the Osteoclast*. pp 290–314. CRC Press: Boca Raton, Florida

Drummond A, Beckett P, Bone et al (1995) BB-2516: an orally bioavailable matrix metalloproteinase inhibitor with efficacy in animal cancer models. *Proc Am Assoc Cancer Res* **36**: 100 (Abstract)

Frey C, Twomey P, Keehn R, Elliot D and Higgins G (1980) Randomized study of 5-FU and CCNU in pancreatic cancer: Report of the veterans administration surgical adjuvant cancer chemotherapy study group. *Cancer* 27–31

Glimelius B, Hoffman K, Sjoden P-O, Jacobsson G, Sellstrom H, Enander L-K, Linne T and Svensson C (1996) Chemotherapy improves survival and quality of life in advanced pancreatic and biliary cancer. *Ann Oncol* **7**: 593–600

Greene J, Wang M, Liu YE, Raymond LA, Rosen C and Shi YE (1996) Molecular cloning and characterisation of human tissue inhibitor of metalloproteinase 4. *J Biol Chem* **271**: 30375–30380

Gress TM, Muller-Pillasch F, Lerch MM, Freiss H, Buchler M and Alder G (1995) Expression and in-situ localisation of gene coding for extracellular matrix proteins and extracellular matrix degrading proteases in pancreatic cancer. *Int J cancer* **62**: 407–413

Harris E (1990) Rhematoid arthritis: pathophysiology and implications for therapy. *N Engl J Med* **322**: 1277–1289

Jones L, Ghaneh P, Humphreys M and Neoptolemos JP (1999) The matrix metalloproteinases and their inhibitors in the treatment of pancreatic cancer. *Ann N Y Acad Sci* **880**: 288–307

Jones LE, Campbell F and Neoptolemos JP (2000) The expression of matrix metalloprotinases (MMPs 1, 2, 3, 7, 8, 9, 12 and 14) and their inhibitors (TIMP 1, 2 and 3) in pancreatic adenocarcinoma. *Br J Surg* **87**: 676

Liotta LA and Stetler-Stevenson WG (1991) Tumour invasion and metastasis: An imbalance of positive and negative regulation. *Cancer Res* **51**(**Suppl**): 5054–5059

Mallinson CN, Rake MD, Cocking JB, Fox CA, Cwynarski MT, Diffey BL, Jackson GA, Hanley J and Wass VJ (1980) Chemotherapy in pancreatic cancer: results of a controlled prospective randomized multicentre trial. *Br Med J* **281**: 1589–1591

Mori M, Barnard GF, Mimori K, Ueo H, Akiyoshi T and Sugimachi K (1995) Overexpression of matrix metalloproteinase 7 mRNA in human colon carcinomas. *Cancer* **7**(**5**)(**Suppl**): 1516–1519

Nomura H, Sato H, Seiki M, Mai M and Okada Y (1995) Expression of membrane-type matrix metalloproteinase in human gastric carcinomas. *Cancer Res* **55**: 3263–3266

Northern and Yorkshire Cancer Registry Information Service (2000) Cancer treatment policies and their effects on survival: Pancreas. NYCRIS: Leeds, UK. (In press)

Pajouh MS, Nagle RB, Breatynach R, Finch JS, Brawler MK and Bowden GT (1991) Expression of metalloproteinase genes in human prostate cancer. *J Cancer Res Clin Oncol* **117**: 144–150

Palmer KR, Kerr M, Knowles G, Cull A, Carter DC and Leonard RCF (1994) Chemotherapy prolongs survival in inoperable pancreatic carcinoma. *Br J Surg* **81**: 882–885

Porte H, Chastre E, Prevot S, Nordlinger B, Empereur S, Basset P, Chambon P and Gespach C (1995) Neoplastic progression of human colorectal cancer is associated with overexpression of the stromelysin 3 and NM-40/SPARC genes. *Int J Cancer* **64**: 70–75

Sellers A, Murphy G, Meickle MC and Reynolds JJ (1997) Rabbit bone collagenase inhibitor blocks the activity of other neutral metalloproteinases. *Biochem Biophys Res Commun* **87**: 581–587

Smith PF, Hoth DF, Levin B, Karlin DA, MacDonald JS and Wooley PV (1980) 5-Fluorouracil, adriamycin and mitimycin C (FAM) chemotherapy for advanced adenocarcinoma of the pancreas. *Cancer* **46**: 2014–2018

Stetler-Stevenson WG (1989) Tissue inhibitor of metalloproteinase (TIMP 2). *J Biol Chem* **264**: 17374–17378

Uria JA, Ferrando AA, Velasco G, Freije JMP and Lopez-Otin C (1994) Structure and expression in breast tumours of human TIMP 3, a new member of the metalloproteinase inhibitor family. *Cancer Res* **54**: 2091–2094

Wolf C, Chenard MP, Durand de Grossouvre P, Bellocq JP, Chambon P and Basset P (1992) Breast-cancer-associated stromelysin-3 gene is expressed in basal cell carcinoma and during cutaneous wound healing. *J Invest Dermatol* **99**: 870–872

*© 2001 Cancer Research Campaign*
